# Supplementary material for: Preparation of an Antioxidant Assembly Based on a Copolymacrolactone Structure and Erythritol following an Eco-Friendly Strategy
Source: Antioxidants (Basel). 2022 Dec 15;11(12):2471. doi: 10.3390/antiox11122471 (PMC9774145; doi:10.3390/antiox11122471)
Supplement: Supplementary file 1 [file antioxidants-11-02471-s001.zip › antioxidants-2032999-supplementary.pdf]

antioxidants

Supporting Information

S1. Fourier Transform Infrared Spectroscopy

Table S1 presents the most important frequencies and their spectral assignments registered from FT-IR spectra of PEBSA_solution and PEBSA_suspension samples. From both spectra, specific bands of the comonomers and of the synthesized copolymers were put into evidence. Thus, bands corresponding to asymmetric and symmetric stretching vibration and bending vibration assigned to methylene groups are present.

The most intense peak registered in both spectra is assigned to carbonyl (C=O) stretching. The bands assigned to C-O stretching vibration and to C-O-C group asymmetric stretching vibration can also be found in both spectra. In the fingerprint region, we can identify bands specific to C=C and C-C bending vibration. However, the differences that can be noticed are the displacements at the level of some absorption peaks and the modification of intensities. Thus, the C-H stretching vibration appears slightly shifted from 2925 cm^{-1} in case of PEBSA_solution to 2923 cm^{-1} in case of PEBSA_suspension while C-H bending vibration appear naturally higher shifted from 1452 cm^{-1} to 1461 cm^{-1}. Carbonyl bonds specific band also appear slightly shifted to lower values in the case of PEBSA_suspension. Another difference that occurs is the C-O-C peak which is recorded at a slightly higher frequency in PEBSA_suspension spectra. These shifts are attributed to the new intra- and intermolecular hydrogen bonds intervened in case of PEBSA_suspension with higher molecular weight. Also, the increase in the intensity of the bands recorded at 1063 cm^{-1} and 1115 cm^{-1}, bands which in PEBSA_solution case were very low in intensity, can be caused by the overlapping of C-C vibration (specific to both copolymers) with the ionic sulphate RSO_3- vibration specific to sodium dodecyl benzene sulfonate and potentially remained as a residues of surfactant from the PEBSA_suspension synthesis.

The presence of sodium dodecyl benzene sulfonate can also be noticed by the increased intensity of the bands from the fingerprint region of the spectra, specific to the aromatic compounds bending vibration and registered at 918 cm^{-1}, 725 cm^{-1} and 678 cm^{-1}. The increased intensity occurs from the overlapping of the squaric acid C=C bond with the aromatic bonds from sodium dodecyl benzene sulphonate.

Table S1. Registered frequencies and the spectral assignments corresponding to PEBSA_solution and PEBSA_suspension samples resulted from FT - IR Spectroscopy.

Assignment	*PEBSA_solution Registered Frequency, cm^{-1}*	*PEBSA_suspension Registered Frequency, cm^{-1}*
C-H Stretching	2925	2923
C-H Stretching	2852	2852
C=O Stretching	1697	1695
C-H Bending	1452	1461
C-O Bending	1280	1280
C-O Stretching	1230	1228
C-O Stretching	1182	1189
C=C Bending	920	918
C-H Bending	727	725
C=C Bending	681	678

S2. NMR Spectroscopy

Figure S1 illustrates the carbon spectrum for PEBSA_Eryt bioactive compound. In the ^{13}C RMN spectrum, signals for methylene carbon atoms can be found at 72 ppm, signals at 63 ppm are assigned to O-bonded methylene carbon atoms, and signals at 175.6 ppm correspond to carbonyl carbons.

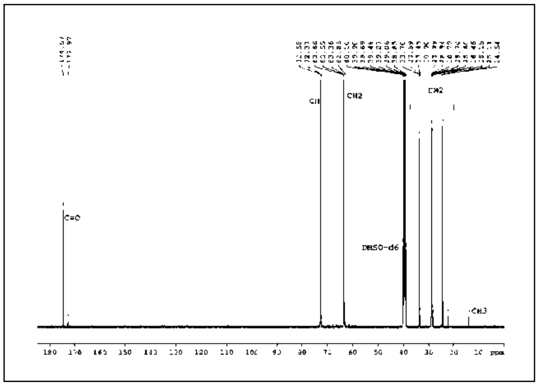

Figure S1. The carbon spectrum for PEBSA_Eryt bioactive compound.

Also, the two-dimensional homonuclear COSY spectra and heteronuclear HSQC and HMBC were recorded and presented (Figures. S2-4).

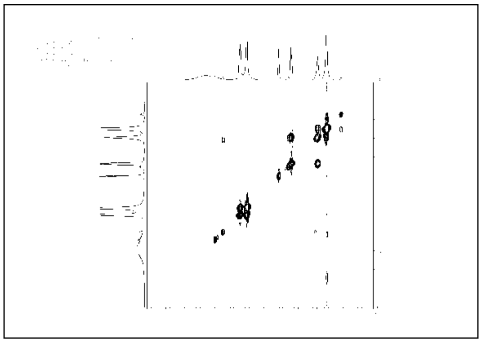

Figure S2. Spectrum H, COSY H for PEBSA_Eryt sample.

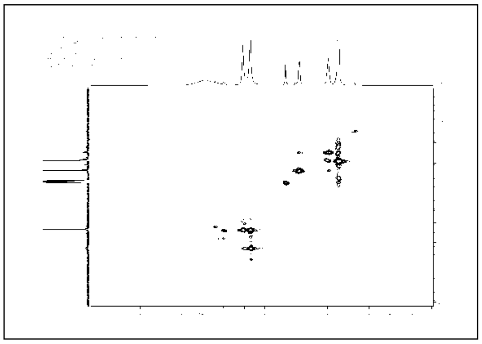

Figure S3. Spectrum H, C HSQC for PEBSA_Eryt.

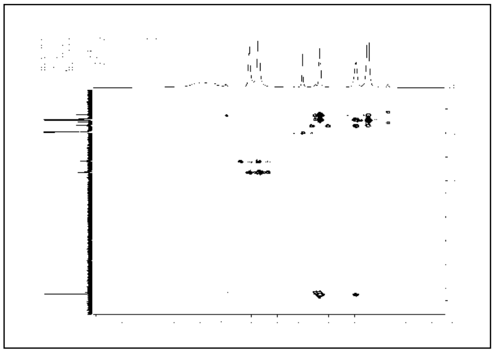

Figure S4. Spectrum H, C HMBC for PEBSA_Eryt.

The data registered from NMR spectroscopy investigation attest the formation and structure of the poly(ethylene brassylate-co-squaric acid) (PEBSA) and PEBSA_Eryt bioactive compound.

S3. Mass Spectrometry Data

The PEBSA_suspension main gases released during the pyrolysis process was also investigated, as STA 449 F1 Jupiter thermobalance is coupled online with mass spectrometer Aëolos QMS 403C (Netzsch) as one system TG-MS. The released gases are transferred through a 290 °C heated quartz capillary (75 mm diameter) at the mass spectrometer. Working parameters of mass spectrometer are: ionization energy with electron impact of 70 eV, vacuum of 10^{-5} mbar, the signals m/z scale from 1 up to 200 amu. The MS spectra were registered and analysed with Aeolos 7.0 software.

Main gases released from PEBSA_suspension during the decomposition process were analysed using mass spectroscopy. Figures S5 a-c present the MS spectra of the released compounds during thermal decomposition of the copolymacrolactone system and extracted at 285°C, 429°C and 455°C temperatures at which the largest amount of gases released were recorded, and are depicted from Gram Schmidt curve**.**

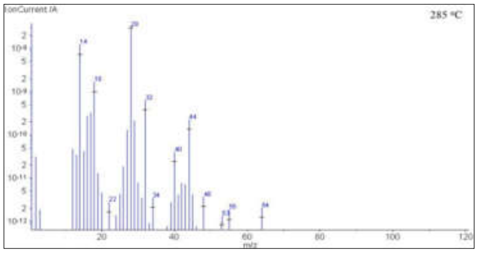

(**a**)

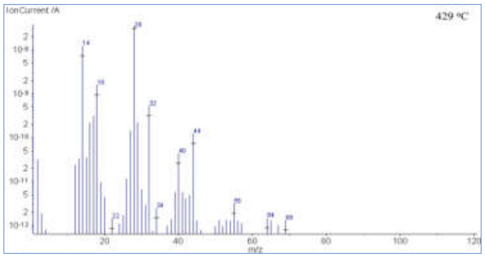

(**b**)

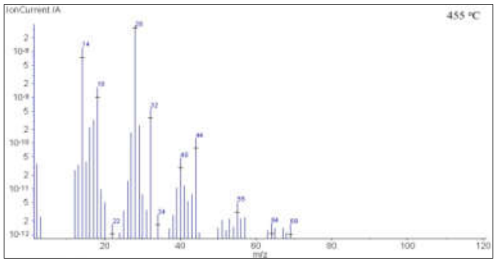

(**c**)

Figure S5. MS spectra of thermal degradation products recorded at 285 °C (**a**), 429 °C (**b**) and 455 °C (**c**).

The identification of the volatile compounds was done using data from NIST MS library [http: webbook.nist.gov/chemistry/ name-ser.html]. The main signals identified are assigned as it follows: water (18, 17, 16 m/z), carbon monoxide (18, 16, 12 m/z), carbon dioxide (44, 28, 22, 16, 12 m/z), formic acid (46, 45, 44, 29, 13 m/z), acetaldehyde (43, 42, 41, 26, 15, 14 m/z), ethane (30, 28, 27 m/z) cyclohexane (69, 56, 55, 41 m/z), acetylene (27, 26, 25, 24 m/z), acetone (58, 43, 42, 38, 26, 27, 14 m/z), butadiene (54, 53, 52, 51, 50, 39 m/z), furan (68, 40, 38, 37 m/z), ethanol (45, 31, 29, 27 m/z), hydrogen sulfide (36, 35, 34, 33, 32 m/z) and SO2 (65, 64, 50, 48, 32, 16 m/z) of the remains sodium dodecylbenzene sulfonate decomposition products. Data are in good agreement with the previous results obtained from the PEBSA_solution decomposition process and attest the formation of the new copolymacrolactone system synthesized by the suspension procedure [1–3].

S4. Antimicrobial Activity

The antimicrobial activity of Eryt at various concentrations (20%, 15%, 10% and 7.5%) was determined by disk diffusion assay against 13 different reference strains: *Staphylococcus aureus* ATCC25923, *Escherichia coli* ATCC25922, *Enterococcus faecalis* ATCC29212, *Klebsiella pneumonie* ATCC10031, *Pseudomonas aeruginosa* ATCC27583, *Salmonella typhimurium* ATCC14028, *Candida albicans* ATCC10231, *Candida albicans* ATCC90028, *Candida glabrata* ATCC20019, *Candida parapsilosis* ATCC2201, *Penicillium chrysogenum* ATC10106, *Cladosporium cladosporioides* ATCC16022, and *Aspergillus brasiliensis* ATCC9642. All microorganisms were stored at -80°C in 20% glycerol. The bacterial strains were refreshed in Mueller-Hinton broth at 37°C, the yeast strains were refreshed on Sabouraud dextrose agar at 37°C, and the fungal strains were refreshed on malt extract agar (MEA) and potato dextrose agar (PDA). Microbial suspensions were prepared with these cultures in sterile solution to obtain turbidity optically comparable to that of 0.5 McFarland standards. Volumes of 0.2 ml from each inoculum were spread onto agar plates. The sterilized paper discs were placed on the plates and an aliquot (15 μl) of the tested concentrations were added on the paper disc. To evaluate the antimicrobial properties, the growth inhibition was measured under standard conditions after 24 hours of incubation at 37 °C for the bacterial and yeast strains and after 72 hours at 25 °C for the fungal strains. All tests were carried out in triplicate to verify the results.

Eryt has already been investigated for its potential to inhibit bacterial growth in dental plaque (mainly for Streptococcus mutans, a gram-positive facultative anaerobic bacterium) studies showing that it can be used successfully in reducing these bacteria. In the case of the present study, we wanted to further investigate the potential of Eryt for antimicrobial applications on other strains to meet the needs of diverse medical applications (mainly drug delivery and regenerative medicine). We concluded that the polyol does not show antimicrobial activity in the case of aerobic strains (Figure S6). Therefore, no additional tests were performed on the PEBSA polymer and the PEBSA_Eryt complex, since the intended applications for the materials under study do not involve their use in anaerobic environments.

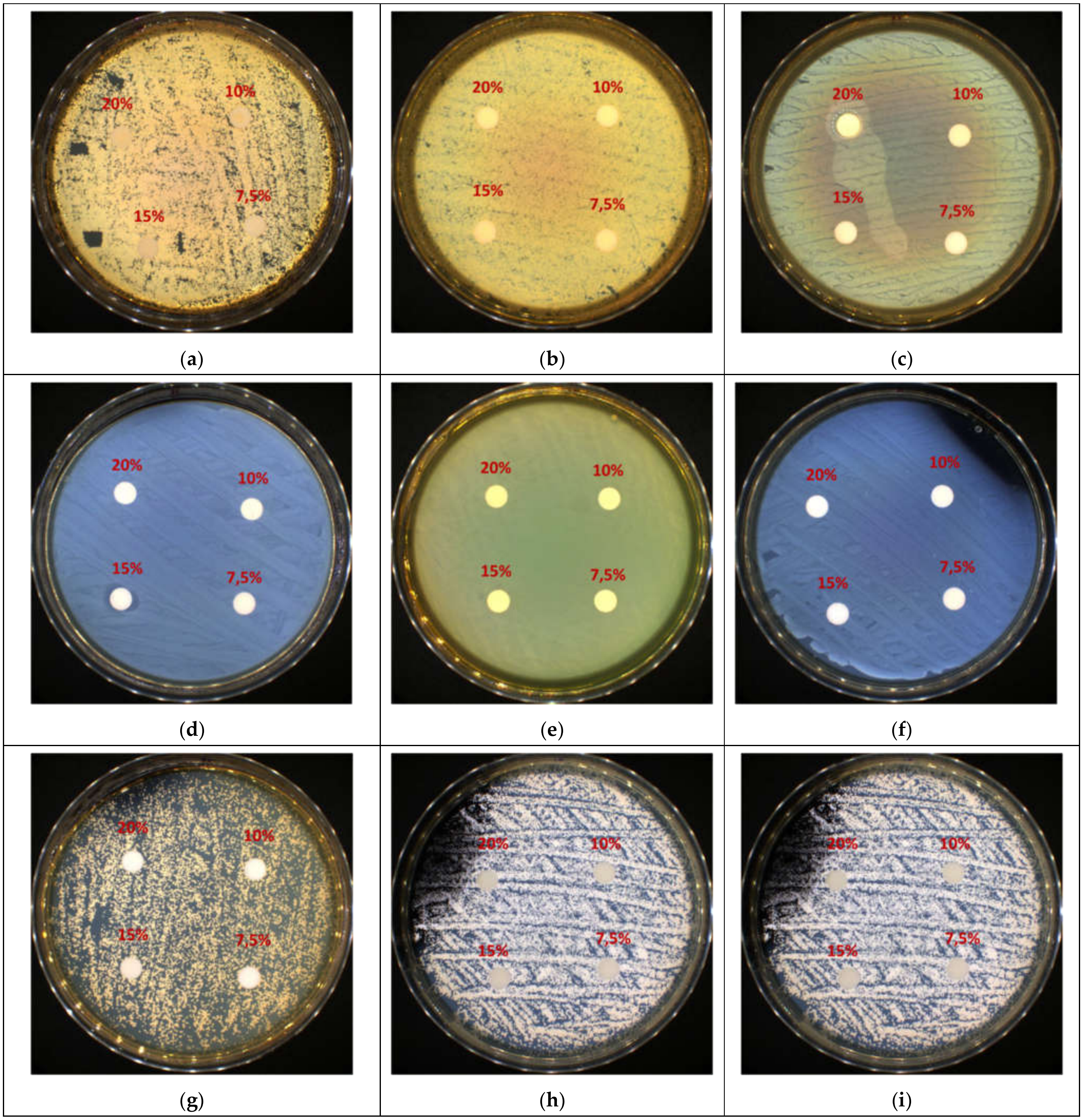

(**a**) (**b**) (**c**)
(**d**) (**e**) (**f**)
(**g**) (**h**) (**i**)

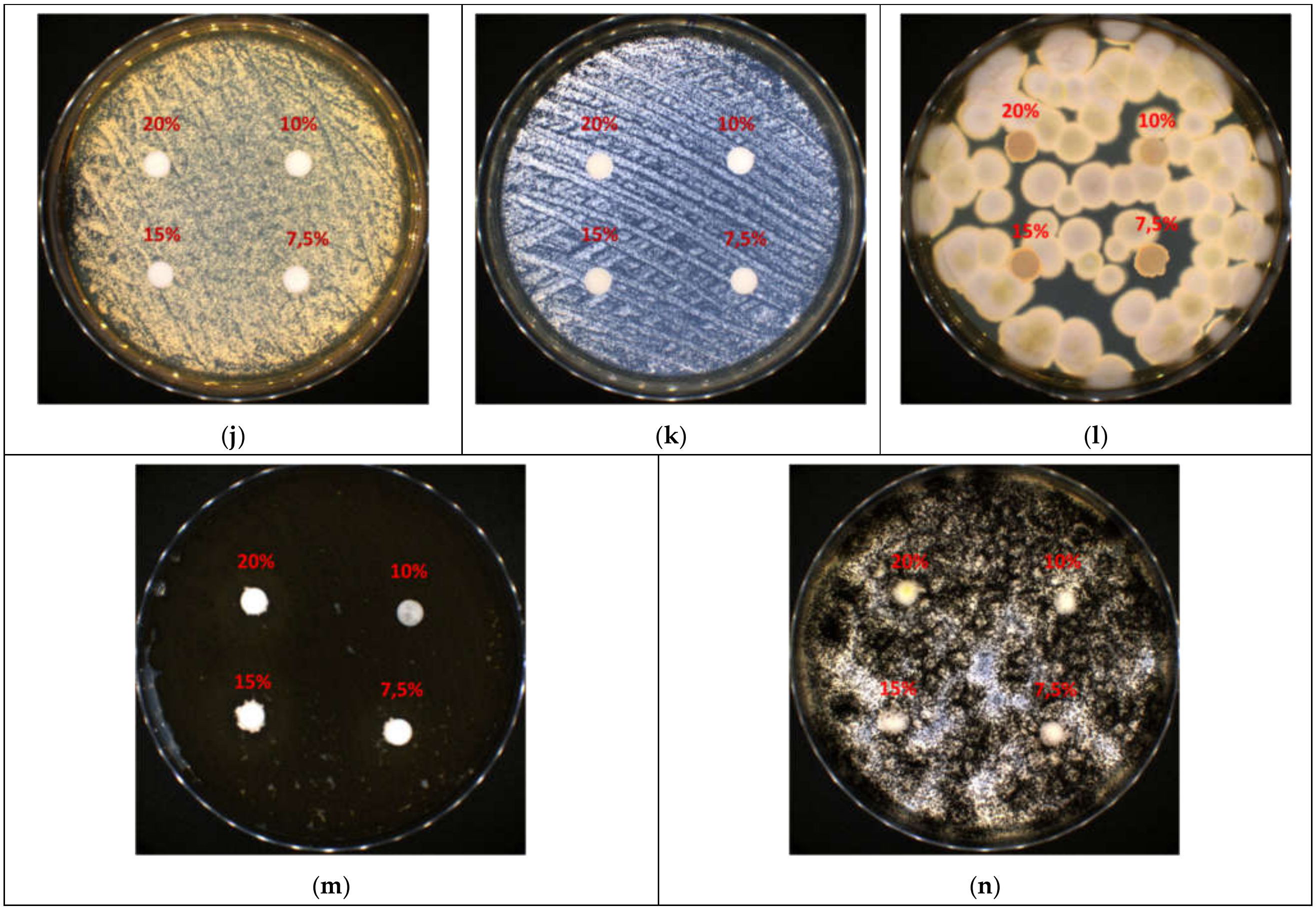

Figure S6. Antibacterial activity of Eryt against (**a**) *Staphylococcus aureus* ATCC25923, (**b**) *Escherichia coli* ATCC25922, (**c**) *Enterococcus faecalis* ATCC29212, (**d**) *Klebsiella pneumonie* ATCC10031, (**e**) *Pseudomonas aeruginosa* ATCC27583, (**f**) *Salmonella typhimurium* ATCC14028, (**g**) *Candida albicans* ATCC10231, (**h**) *Candida albicans* ATCC10231, (**i**) *Candida albicans* ATCC90028, (**j**) *Candida glabrata* ATCC20019, (**k**) *Candida parapsilosis* ATCC2201, (**l**) *Penicillium chrysogenum* ATC10106, (**m**) *Cladosporium cladosporioides* ATCC16022, (**n**) *Aspergillus brasiliensis* ATCC9642.

References

1. Chiriac, A.P.; Rusu, A.G.; Nita, L.E.; Macsim A.M.; Tudorachi, N.; Rosca, I.; Doroftei, F. Synthesis of Poly(Ethylene Brassylate-Co-squaric Acid) as Potential Essential Oil Carrier. *Pharmaceutics* **2021**, *13*, 477.
2. Jakab, E.; Mészáros, E.; Omastová, M. Thermal decomposition of polypyrroles. J. Therm. Anal. Calorim. 2007, 88(2), 515–521.
3. Chiriac, A.P.; Asandulesa, M.; Stoica, I.; Tudorachi, N.; Rusu, A.G.; Nita, L.E.; Chiriac, V.M.; Timpu, D. Comparative study on the properties of a bio-based copolymacrolactone system. *Polym. Test.* **2022**, *109*, 107555.
